# Nanomolecular silencing of TSC22D4 mRNA via a DNAsome-siRNA for enhancing insulin sensitization in hepatocytes

**DOI:** 10.22038/ijbms.2024.81998.17744

**Published:** 2025

**Authors:** Ameneh Mohammadi, Pedram Ebrahimnejad, Said Abediankenari, Zahra Kashi, Pooria Gill

**Affiliations:** 1 Department of Pharmaceutics, Faculty of Pharmacy, Mazandaran University of Medical Sciences, Sari, Iran; 2 Pharmaceutical Sciences Research Center, Hemoglobinopathy Institute, Mazandaran University of Medical Sciences, Sari, Iran; 3 Research Center of Immunogenetics, Mazandaran University of Medical Sciences, Sari, Iran; 4 Research Center for Diabetes, Mazandaran University of Medical Sciences, Sari, Iran; 5 The Health of Plant and Livestock Products Research Center, Mazandaran University of Medical Sciences, Sari, Iran; 6 Department of Medical Nanotechnology, Faculty of Advanced Technologies in Medicine, Mazandaran University of Medical Sciences, Sari, Iran

**Keywords:** DNAsome, HepG2, Insulin resistance, siRNA, TSC22D4

## Abstract

**Objective(s):**

Insulin resistance (IR) is a critical component of metabolic syndrome, primarily linked to obesity. It contributes to impaired glucose metabolism, beta-cell dysfunction, and the onset of type 2 diabetes. This study aimed to develop a DNAsome nanocarrier designed for the targeted delivery of small interfering RNA (siRNA) to inhibit mRNA of Transforming growth factor beta-like Stimulated Clone 22 D4 (TSC22D4), thereby enhancing insulin sensitivity in hepatocytes.

**Materials and Methods:**

The DNAsome was constructed using Y-DNA building blocks derived from three distinct DNA oligonucleotides. Its structural characteristics were analyzed through atomic force microscopy (AFM). The functional efficacy of the DNAsome in delivering siRNA was evaluated by measuring its cellular uptake and ability to down-regulate TSC22D4 expression in HepG2 cells via real-time PCR. Additionally, the cytotoxicity and safety of both the DNAsome and the DNAsome-siRNA complexes were assessed using the MTT assay on HepG2 cells.

**Results:**

Findings indicated successful fabrication of the DNAsome nanocarriers, although aggregation was observed at higher concentrations, yielding nanoparticle sizes between 116 and 740 nm. Real-time PCR results confirmed effective siRNA targeting, significant cellular uptake of the nanocomplexes, and successful silencing of TSC22D4 expression.

**Conclusion:**

This study suggests that DNAsome-based siRNA delivery systems hold promise for improving insulin sensitivity and addressing IR associated with obesity and metabolic syndrome.

## Introduction

Excessive lipid storage combined with reduced removal in humans leads to overweight and various related health conditions, including insulin resistance, cardiovascular complications, and dyslipidemia. This global health issue currently affects an alarming population of over 1.5 billion individuals worldwide (1, 2). The main characteristic of the metabolic syndrome is insulin resistance, which eventually results in metabolic dysfunction, including glucose intolerance, pancreatic beta cell loss, and finally, type 2 diabetes (3).

Numerous pathways may be involved in the onset and development of type 2 diabetes. The three main abnormalities are reduced glucose absorption in peripheral muscles, increased glucose synthesis in the liver, and beta-cell failure. In addition, other pathogenic mechanisms can also play a role, such as insulin resistance in adipocytes, leading to increased lipolysis, reduced secretion or sensitivity of incretin in the gastrointestinal tract, increased glucagon secretion from alpha cells, increased glucose reabsorption in the kidneys, and insulin resistance in the central nervous system as a result of neurotransmitter dysfunction in the brain (4).

The development of diabetes and the need for pharmaceutical intervention to avoid late complications are significantly influenced by the insensitivity of essential metabolic organs to insulin action, including the liver, skeletal muscle, and adipose tissue. Therefore, a desired goal of diabetes management still involves achieving effective and secure insulin sensitivity. Sulfonylureas, metformin, thiazolidinediones, alpha-glucosidase inhibitors, incretin mimetics, and dipeptidyl-peptidase - 4 inhibitors are the main groups of medications used for anti-diabetic and/or insulin-sensitizing reasons. Meanwhile, these medications come with significant restrictions (5). Transcriptional co-factor complexes have been recognized as crucial regulators coordinating metabolic programs across various tissues, such as the liver and white adipose tissue (6-8).

The Transforming growth factor beta-like Stimulated Clone (TSC) 22 D4 is critical in regulating insulin signaling and glucose metabolism. In mouse models of diabetes, inhibiting the activity of TSC22D4 in the liver can prevent and reverse hyperglycemia, glucose intolerance, and insulin resistance. TSC22D4 affects glucose homeostasis by directly controlling the expression of Lipocalin 13 (LCN13), a small protein secreted from cells. Notably, patients with diabetes show elevated levels of TSC22D4 expression in the liver, linked to reduced insulin sensitivity, high blood sugar levels, and lower LCN13 serum concentrations (9). Given the issues with efficacy and safety, as well as limitations associated with insulin-sensitizing drugs, alternative approaches to treatment, such as gene therapy, may offer more promising prospects for improving patient outcomes. Molecular-targeted therapeutics involve purposefully changing a gene expression to cure pathological diseases. Exogenous nucleic acids, such as DNA, mRNA, siRNA, miRNA, or antisense oligonucleotides, are introduced to cause this change (10, 11). RNA-based gene therapy must function within target cells to treat diseases without inducing unwanted immune responses (12). To deliver encoding gene sequences precisely to target tissues and enable therapeutic protein expression in diseased cells, Nonviral drug delivery systems are a promising alternative to viral vectors for delivering RNA into cells (13, 14). These systems provide new disease prevention and therapy options since they often rely on synthetic carriers, including polymeric materials, liposomes, and hydrogels (15-19). Specific nonviral systems can concurrently deliver numerous medications and/or medications containing nucleic acids (20, 21). However, the isotropic and polydisperse nature of most polymeric materials and liposomes makes it challenging to engineer building blocks with multiple functionalities for tailored multidrug delivery (22, 23). Current gene delivery methods must be more efficient and improved to achieve clinical utility. Fortunately, anisotropic building blocks and multifunctional DNA nanostructures have been created due to DNA’s chemical recognition abilities (24-26).

DNA nanotechnology has the potential to advance biomedical engineering by providing new treatments and diagnostic tests. DNA nanostructures can be customized with different chemical and biological substances at the nanometer level, making them suitable for precise diagnostic instruments and carriers for targeted medication administration (27). They can also be used to create dynamic devices, such as DNA nanorobots, that can perform programmed activities and respond to environmental stimuli with precise control over their form, size, and function (28). In this study, we employed a nanomolecular approach to developing a DNA-based nanocarrier termed DNAsome, consisting of a DNA-based liposome-like core-shell structure. This nanocarrier was fabricated using branched DNA-lipid hybrid molecules as its foundational components, allowing for highly efficient and targeted silencing of siRNAs in liver cells (hepatocytes) (29). By incorporating these hybrid molecules into the core-shell structure of DNAsome, we achieved enhanced stability, improved cellular uptake, and nanomolecular silencing of hepatocytes. The primary objective of our research was to achieve targeted silencing of the TSC22D4 gene, thereby enhancing hepatocellular insulin sensitization. 

## Materials and Methods

### Ethical statement

The project was found to be based on ethical principles and national norms and standards for conducting medical research in Iran (I.R.MAZUMS.IMAMHOSPITAL.REC.1397.110).

### DNAsome nanocarrier assembly

Branched DNAs are formed by three separate single-stranded DNA (ssDNA) (Table 1), which can be assembled into a DNAsome nanoscale structure by placing them in a buffer solution and adjusting the pH.

DNA oligonucleotides were purchased from BioNeer South Korea. Y1 is a sequence with a hydrophobic section attached (Cholesteryl-TEG (triethylene glycol)). Y2 is a sequence containing the complementary siRNA section, and Y3 is a complementary section for the structure of Y-DNA. DNAs oligonucleotides (ssDNA) were dissolved in a buffer (1X = Trys 10 mM –EDTA 1 mM –NaCl 50 mM) pH=8, in four concentrations: 10, 20, 40, and 80 nM, and hybridization was performed under the following temperature program: (1) Denaturation at 95 °C for 2 min. (2) Cooling at 65 °C and incubation for 2 min. (3) Annealing at 60 °C for 5 min. (4) Further annealing at 60 °C for 0.5 min with a continuous temperature decrease at 1 °C per min. The final products containing DNA nanostructures with 10, 20, 40, and 80 nM concentrations were analyzed by Dynamic Light scattering (DLS) and atomic force microscopy (AFM) imaging.

### Atomic force microscopy of DNAsome nanocarriers

AFM was operated in contact mode with JPK-AFM, with 150 Hz IGain, 0.0048PGain, and 1.0 V set point via a JPK NanoWizard control. The cantilever was ACTA-10 probe model (material: silicon, N-type, 0.01–0.025 Ω/cm). The preliminary data was analyzed graphically using the JPK Nanoanalyzer software (30).

### Dynamic light scattering and Zeta potential analysis of DNAsome nanocarriers

To confirm the fabrication of nanoscaled DNAsomes with suitable surface chemistry for effective interaction with hepatocytes while encapsulating siRNAs, we conducted measurements of dynamic light scattering and Zeta potential. The assembled DNA nanocarrier was diluted in PBS buffer (pH 8), and we utilized the ZetaSizer Nano ZS system (Malvern Instruments, Worcestershire, UK) to obtain data on Zeta potential, mean particle size, and size distribution.

### Culture of HepG2 cell line

Cell culture analysis was performed using human liver hepatocellular carcinoma HepG2 was purchased from the Iranian Biological Resource Center. The cells were cultured in Dulbecco’s Modified Eagle Medium/Nutrient Mixture F-12 (DMEM/F-12) supplemented with 10% fetal bovine serum (FBS) and 1% penicillin/streptomycin at 37^o^C in 5% CO_2_ according to the manufacturer’s instructions. Also, cells were subcultured every 4–5 days using Trypsin/EDTA.

### Cell line treatment with DNAsome nanocarrie

For the transfection study, the TSC22D4 specific siRNA (9) (5’- GGACGUGUGUGGAUGUUUAdTdT-3’) was purchased from BioNeer South Korea, loaded onto DNAsome nanocarrier. The DNAsome at the sticky end of the Y-DNA was hybridized with the single-stranded siRNA. DNAsomes and single-stranded siRNA had a molar ratio of 1:1. Then, HepG2 cells were seeded on a 48-well plate at a density of 2 × 10 ^4 ^cells per well, and DNAsome/DNAsome-siRNA complex was added simultaneously 5 µl for each concentration. The cells were incubated at 37 °C for 4 hr. Then, the supernatants were removed, and fresh culture medium was added to the cells, which were then incubated again for 48 hr.

### Real-time PCR for investigating TSC22D4 gene expression changes

At the end of the time, cells were washed once with PBS lysis. Total RNA was extracted and purified from infected cells using RNA extraction kit buffers (DENAzist column RNA isolation kit) as the kit’s protocol. cDNA was prepared and amplified by reverse transcription using one-pot Easy cDNA Synthesis Kit ParsTus, containing H-minus MMLV (Thermostable) enzyme and random hexamer primers. Quantitative real-time RT-PCR was used to evaluate the expression of human TSC22D4 mRNA in a fluorescent temperature cycler using the SYBR Green assay, and fluorescence was detected on StepOne software (Applied Biosystems | Thermo Fisher Scientific). The real-time PCR was performed in a 20 µl reaction volume using a SYBR Green Real-Time kit (2X SYBR® Green Real-Time PCR Master Mix kit, Parstous, Iran). One pmol of primers and 100 ng total RNA-equivalent of each cDNA generated by reverse transcription were used. Primers for TSC22D4 targeted sequences and housekeeping gene β-Actin are reported in Table 2 (29). The following thermal cycling profile was used: 94 ^o^C for 60 sec, followed by 35 cycles of 95 ^o^C for 15 sec, 55 ^o^C for 30 sec, and 72 ^o^C for 60 sec. Human TSC22D4 mRNA expression was evaluated by ΔΔCt calculation. 

### MTT assay

Cell viability MTT assay assessed the cytotoxicity of the DNAsome and siRNA hybridized DNAsome. HepG2 cells were seeded in a 96-well plate at 10^ 4 ^cells per well concentration and incubated for 24 hr at 37 °C and CO_2_ (5%). Next, 5 µl of DNAsome and siRNA hybridized DNAsome and fresh media were added to cells at different concentrations and incubated further for 48 hr. Then, 20 µl MTT solution (5 mg/ml), filtered by a 0.22-micron syringe filter, was added to cells and incubated for four hours. The supernatant was removed, and 100 µl DMSO was added to each well to dissolve the formazan crystals. The absorbance was measured spectrophotometrically using an Elisa microplate spectrophotometer at 570 nm. All absorbance values were corrected against blank wells. The cell viability was calculated by the following formula (A = absorbance):

**Equation 1:** Cell viability (%) = (A sample – A blank) / (A control – A blank) × 100

### AFM-based topographic characterization of treated and untreated cells with DNAsome-siRNA complex

AFM imaging of cells was performed before and after treatment with DNAsome-siRNA complex to investigate cellular topology changes related to surface receptor expression changes. Three imaging methods were used for this purpose (30). HepG2 cells were seeded on a specialized AFM cell and tissue culture dish and incubated for 24 hr at 37 °C, with 5% CO_2_ in the incubator to allow the cells to adhere to the dish. Then, the cell culture medium was removed, and the cells were washed three times with PBS. Next, a small amount of liquid nitrogen was added to the cell dish and allowed to evaporate completely.

Further, cells were fixed. The cell culture medium was replaced for imaging from the treated HepG2 cells with DNAsome-siRNA complex, 10 microliters of 10 nM nanocarrier were added to the cells, and the dish was incubated again for 48 hr. After 48 hr, the cell culture medium was removed, and the cell dish was washed thrice with PBS. Next, a small amount of liquid nitrogen was added to the cell dish and allowed to evaporate completely. All the AFMs were performed in contact mode using an ACTA-10 model probe of N-type silicon with a resistance of 0.01-0.025 Ω/cm. Subsequently, the images were analyzed utilizing the JPK Nanoanalyzer software.

## Results

### Confirmation of Y-DNA self-assembly reaction and the formation of nanocarrier

**Figure 1:**
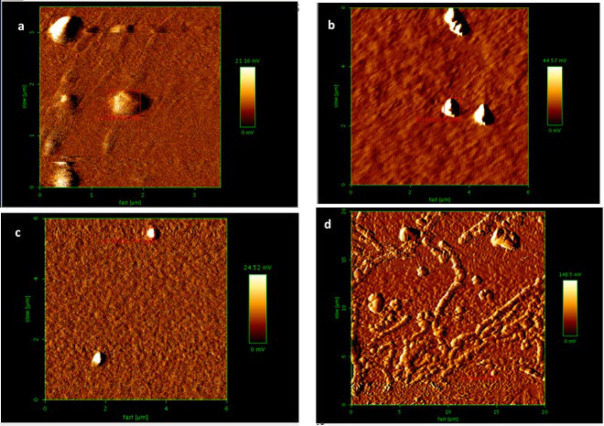
AFM micrographs of the assembled DNAsomes with 10 nM (a), 20 nM (b), 40 nM (c), and 80 nM (d) of the oligonucleotides for the Y-DNA building blocks

We conducted several experiments to validate the self-assembly reaction of Y-DNA and the formation of DNAsome nanocarriers. Initially, we examined the self-assembly reaction of Y-DNA at various concentrations. As a negative control, we repeated the reaction using oligonucleotides lacking the hydrophobic part, confirming that nanocarrier formation did not occur without the lipophilic moiety. This control experiment served as a crucial validation step, proving the necessity of the hydrophobic component for the formation of DNAsome nanocarriers. Atomic force microscopy (AFM) imaging was employed to visualize the formation of DNAsome nanocarriers. The imaging analysis confirmed the presence of DNAsome nanocarriers at concentrations of 10, 20, 40, and 80 nM. The particles displayed sizes ranging from 290 to 740 nanometers, as depicted in Figure 1. It was worth noting that at the highest concentration tested (80 nM), the nanoparticles tended to aggregate and stick together (i.e., malformations), potentially due to their increased densities in particulation. Furthermore, to validate the specificity of DNAsome formation further, we performed imaging of the adverse control reaction. This control experiment was designed to investigate and confirm particle formation’s absence in the hydrophobic moiety’s absence. 

### Particle size and zeta-potential measurements

 Following confirmation of the assembled DNAsomes by AFM, DLS, and Zeta-potential analyses were employed to fully characterize and determine the surface charges, sizes, and PDIs of the DNAsomes. Due to the measurement of a more significant number of particles compared to AFM, the recorded size results differed via DLS. The results revealed that the average sizes of the particles were 362 ± 15, 507 ± 21, and 740 ± 24 nm at the concentrations of 10, 20, and 40 nM, consequently; moreover, the zeta potentials of the DNAsomes were approximately -19.5 ± 1.1, -24.3 ± 0.76, and -39.7 ± 1.2 mV at these concentrations (Table 3). It should be noted that due to the deformity of the nanoparticles had been demonstrated by AFM at a concentration of 80 nM Y-DNA, the measurement of these parameters was not examined.

### TSC22D4 gene expression changes using real-time PCR

**Figure 2:**
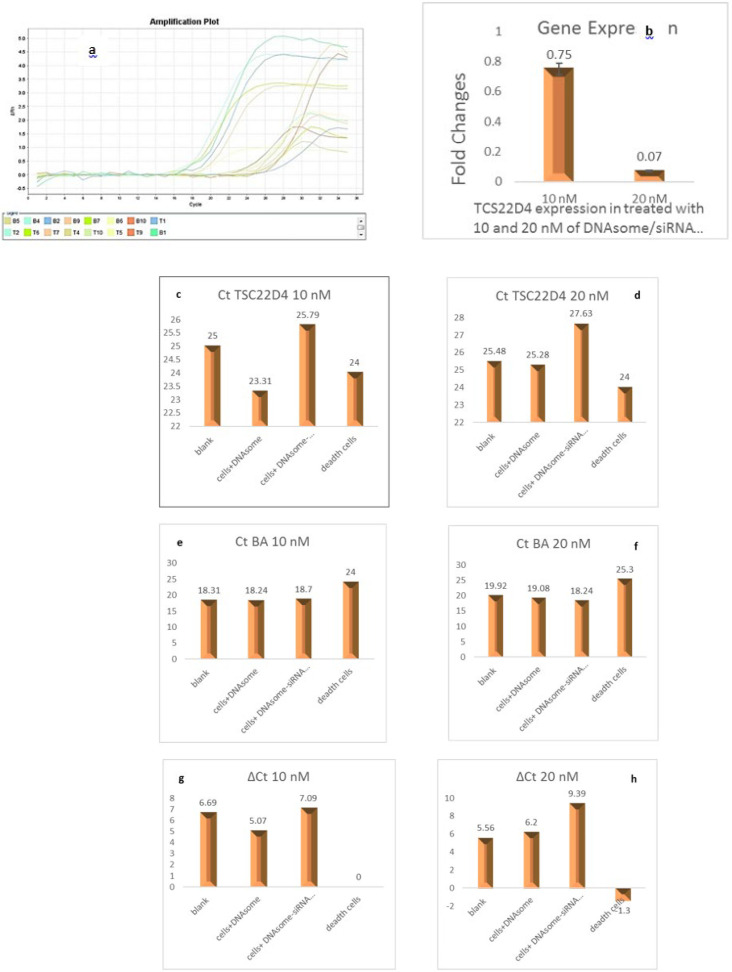
a) Real-time PCR plots; b) Comparison of TSC22D4 Fold Change: Relative measurement of TSC22D4 expression levels after treating HepG2 cells with DNAsome-siRNA complexes at 10 and 20 nanomolar concentrations; The Fold Change in TSC22D4 mRNA at concentrations of 10 and 20 nanomolar decreased by 0.75 and 0.07, respectively, compared to control cells that received no treatment (*P*<0.05); c) Ct calculation of TSC22D4 in 10 nM concentration of DNAsome nanocarrier/DNAsome-siRNA complex; d) Ct calculation of TSC22D4 in 20 nM concentration of DNAsome nanocarrier/DNAsome-siRNA complex; e) Ct calculation of beta-actin (BA) in 10 nM concentration of DNAsome nanocarrier/DNAsome-siRNA complex; f) Ct calculation of beta-actin (BA) in 20 nM concentration of DNAsome nanocarrier/DNAsome-siRNA complex; g) ΔCt calculation for 10 nM of DNAsome nanocarrier/DNAsome-siRNA complex; h) ΔCt calculation for 10 nM of DNAsome nanocarrier/DNAsome-siRNA complex

Real-time PCR data analysis was conducted using the comparative ΔΔCt method to evaluate the mRNA expression of the target gene TSC22D4 in two experimental groups treated with DNAsome/siRNA nanocarrier complexes at concentrations of 10 and 20 nM. The results demonstrated a significant and dose-dependent reduction in TSC22D4 mRNA expression compared to the control cells that received no treatment (*P*<0.05). The Fold Change for TSC22D4 mRNA at 10 and 20 nM concentrations exhibited decreases of 0.75 and 0.07, respectively, signifying the effective delivery of specific siRNA for TSC22D4 mRNA into the cells by the DNAsome nanocarrier (Figure 2, Table 4). 

### Toxic effects of nanocarrier/SiRNA nanocarrier complex on HepG2 cell line

**Figure 3:**
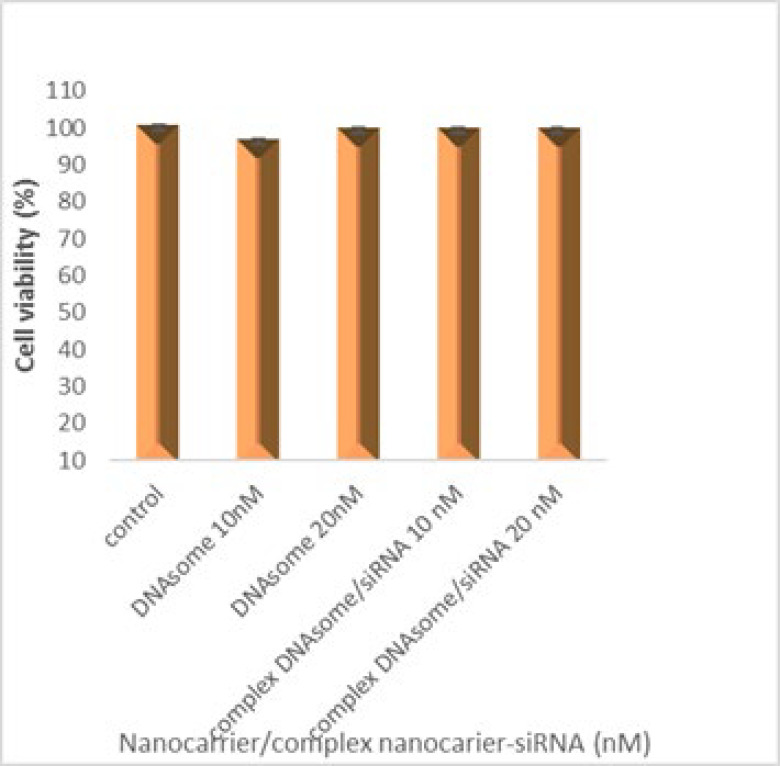
Cell viability as determined by MTT assay in the function of DNAsome nanocarriers and DNAsome/siRNA complexes at 10 and 20 nM concentrations; Cell survival in the samples treated with nanocarrier/siRNA complexes at 10 and 20 nM concentrations was 99.3% and 99.2%, respectively (*P*>0.05).

To assess the toxic effects of the nanocarrier/siRNA complex on the HepG2 cell line, we tested the nanocarrier without siRNA and the nanocarrier/siRNA complex at concentrations of 10 and 20 nanomolar on the cells for 48 hr. The results showed that the percentage of cell survival in the samples treated with nanocarrier at 10 and 20 nanomolar concentrations was 96.1% and 99.1%, respectively. On the other hand, the cell survival in the samples treated with nanocarrier/siRNA complex at 10 and 20 nanomolar concentrations was 99.3% and 99.2% (Figure 3).

### Alterations in cellular topology pre- and post-treatment of HepG2 cells with DNAsome/siRNA complex

**Figure 4:**
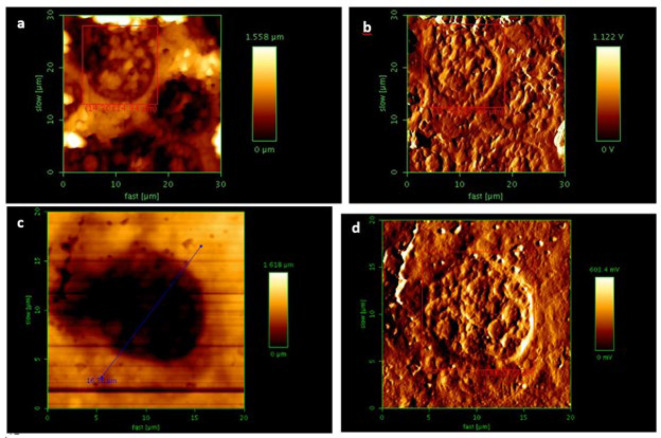
AFM micrographs of N2 fixed treated and untreated HepG2 cells

In this study, cell membrane roughness was measured by using AFM (Figure 4). Ra (Average Roughness), RMS Roughness or Rq, and Peak to Valley Roughness, Rt, have been measured. Based on the data from Table 5, it was evident that after the cellular treatment with DNAsome-siRNA complexes, Ra increased by more than two-fold, and Rq almost doubled. Roughness calculations were performed automatically by AFM analyzing software.

## Discussion

This study focuses on forming a biocompatible nanocarrier and evaluating its performance for siRNA delivery. While the use of liposomal and niosomal drug delivery systems (31-34) is currently under development, and compared to the viral vectors that are used for gene therapy (35), the DNAsome drug delivery system is being investigated here as an efficient, safe and biocompatible system (36). Moreover, they offer the advantage of achieving the synergistic and simultaneous effects of co-delivery of genes and drugs (37). DNAsome is a co-carrier because its amphiphilic structure can carry hydrophilic and lipophilic drugs. DNAsome is a multifunctional nanocarrier that can modify DNA groups (38).

These experimental results strongly confirm the Y-DNA self-assembly reaction and the formation of DNAsome nanocarriers. The AFM imaging analysis not only demonstrated the presence of suitably sized nanocarriers but also revealed the impact of concentration on the aggregation behavior of the nanoparticles. The negative control experiment also solidified our design approach’s validity and affirmed the hydrophobic moiety’s crucial role in facilitating DNAsome formation. These findings lay the foundation for further investigations into the functionality and efficacy of DNAsome nanocarriers for targeted gene delivery and therapeutic applications.

Upon examining the DLS results, it was noted that the nanocarrier, composed of DNA, exhibited a negative zeta potential as anticipated. Notably, the average size of the nanowire increased with the variation in Y-DNA concentration, highlighting a significant observation. This suggests that as the concentration of amphiphilic DNA increases, the zeta potential and size of DNA nanoparticles also increase, aligning with the findings of Roh and colleagues (39).

The real-time PCR findings provide compelling evidence for the ability of the DNAsome nanocarrier to successfully deliver siRNA molecules and trigger the suppression of the target gene TSC22D4. The obtained results demonstrated more efficiency of DNAsomes in achieving gene silencing with a significant reduction in TSC22D4 expression at 20 nM concentration of Y-DNAs in comparison to the 10 nM concentration. Importantly, our results aligned with previous studies, further supporting the effectiveness and consistency of the DNAsome nanocarrier in gene delivery and silencing. The observed reduction in TSC22D4 mRNA expression substantiates the specificity of siRNA targeting, confirming the direct impact of the DNAsome nanocarrier in facilitating the suppression of the TSC22D4 gene. These findings underscore the immense potential of the DNAsome nanocarrier as a reliable and efficient platform for targeted gene therapy. The ability to precisely deliver siRNA against TSC22D4 mRNA and achieve significant gene silencing represents a crucial step toward enhancing insulin sensitivity in liver cells. Our study confirms and supports the effects of TSC22D4 siRNA found in the survey conducted by Ekim Üstünel *et al.* (9). 

According to the cytotoxicity study results, neither the DNAsome nanocarrier nor siRNA-DNAsome complex had a significant toxic effect on liver cells at the examined concentrations (10 nM and 20 nM concentrations), compared to cells that did not receive any treatment. Given the biocompatible nature of the nanocarrier, which is made of DNA, these findings were expected. 

Membrane roughness is vital in examining nanoparticle absorption by cells (40). In this study, an attempt has been made to explore the relationship between the cell membrane roughness and changes in the expression level of some cellular receptors, suppressing TSC22D4 expression (9). Ra (average roughness) calculates the arithmetic mean of absolute coordinate values of roughness, which is one of the most critical factors in determining surface roughness and provides a good description of changes in surface height. RMS Roughness stands for Root-Mean-Square, which is essentially the mean deviation and is used in statistical calculations. RMS Roughness or Rq is the root mean square roughness in roughness specifications. Peak-to-valley roughness, denoted by Rt in surface roughness calculations, is the difference in height between the highest point or peak and the deepest point or valley, meaning the difference between the highest peak and the deepest valley measured on the surface. The AFM-based nanoscopic analysis of the cell membrane reveals significant changes before and after exposure to the DNAsome-siRNA complex (Ra and Rq increased about two-fold). This increase might be attributed to increased expression of some insulin-sensitive receptors (e.g., lipocalin 13 receptor) (9). Since cell roughness increases during nanoparticle absorption, this increase in hardness can be attributed to cellular uptake. The cellular uptake of nanoparticles, facilitated by endocytosis (40), highlights the direct correlation between enhanced surface roughness and the uptake of the nanoparticles.

**Table 1:** Oligonucleotide sequences of the Y-DNA building blocks in DNAsome nanocarriers

Y1	5' - /NH_2_/ CTT ACG GCG AAT GTC ATG CGG ATC CA - 3'
Y2	5' - AAT AAA CAT CCACAC AGC TCC AGG CTG ATT CGG TCA TTC GCC GTA AG -3'
Y3	5' - TGG ATC CGC ATG AAC CGA ATC AGC CT - 3'

**Table 2:** Oligonucleotide sequences of forward and reverse primers for TSC22D4 and β- A

Gene	Forward primer 5'>3'	Reverse primer 5'>3'
TSC22D4	TGCGGATGGAGTTGGGTGCT	TGGAGATTTGTGAACCAGGC
-ACTINβ	GCACTCTTCCAGCCTTCCTT	CGTACAGGTCTTTGCGGATG

TSC22D4: Transforming growth factor beta-like Stimulated Clone 22 D4

**Table 3:** Zeta potential and size of DNAsomes in different concentrations of Y-DNA

Concentration of Y-DNA (nM)	Zeta potential (mV)	Average particle size (nm)	Polydispersity index (PDI)
10	-19.5 ± 1.1	362 ± 15	0.5
20	-24.3 ± 0.7	507 ±21	0.5
40	-39.7 ± 1.2	740 ± 24	0.7

a and b) N2-fixed untreated HepG2 cell; c and d) N2-fixed treated HepG2 cell with DNAsome-siRNA complex

**Table 5:** Parameters measured by AFM in N2 fixed untreated and treated HepG2 cells

Parameter	N_2_ fixed untreated HepG2 cell	N_2_ fixed treated HepG2 cell
Average Roughness Ra (nm)	136.6	312.4
RMS Roughness Rq (nm)	179.4	345.7
Peak-to-Valley Rt (µm)	1.505	1.247
Length (µm)	14.30	11.88
Width (µm)	14.94	12.58

## Conclusion

In this study, we successfully developed a DNAsome nanocarrier utilizing Y-DNA building blocks through a self-assembly process of oligonucleotides. This nanocarrier was specifically engineered for targeted siRNA delivery against TSC22D4 mRNA, aimed at enhancing insulin sensitivity in liver cells. Atomic force microscopy (AFM) confirmed the formation of appropriately sized nanocarriers, validating the self-assembly method. Real-time PCR analysis demonstrated the nanocarrier/siRNA complex’s remarkable efficiency in siRNA loading, cellular uptake, and gene silencing capabilities. Notably, we observed increased cellular surface roughness, indicating successful nanoparticle absorption and effective gene silencing. Importantly, toxicity studies on liver cell lines showed no adverse effects associated with either the nanocarrier or the complex, with cell survival rates consistently exceeding 96%, underscoring its biocompatibility. Our findings highlight the potential of the DNAsome nanocarrier for safe and effective gene delivery in therapeutic applications. By targeting the TSC22D4 gene specifically, we have demonstrated effective gene expression knockdown and potential improvements in insulin sensitivity in liver cells. This research lays a strong foundation for future advancements in targeted gene therapy and innovative gene delivery systems.

## Data Availability

The authors confirm that the data supporting the findings of this study are available within the article.

## Declaration

We have not used any AI tools or technologies to prepare this manuscript. Also, the final content was thoroughly reviewed by the authors and they are fully responsible for the content of this manuscript.
